# Supplementary figures and images for: Sequences within and upstream of the mouse Ets1 gene drive high level expression in B cells, but are not sufficient for consistent expression in T cells
Source: PLoS One. 2025 Mar 7;20(3):e0308896. doi: 10.1371/journal.pone.0308896 (PMC11888140; doi:10.1371/journal.pone.0308896)

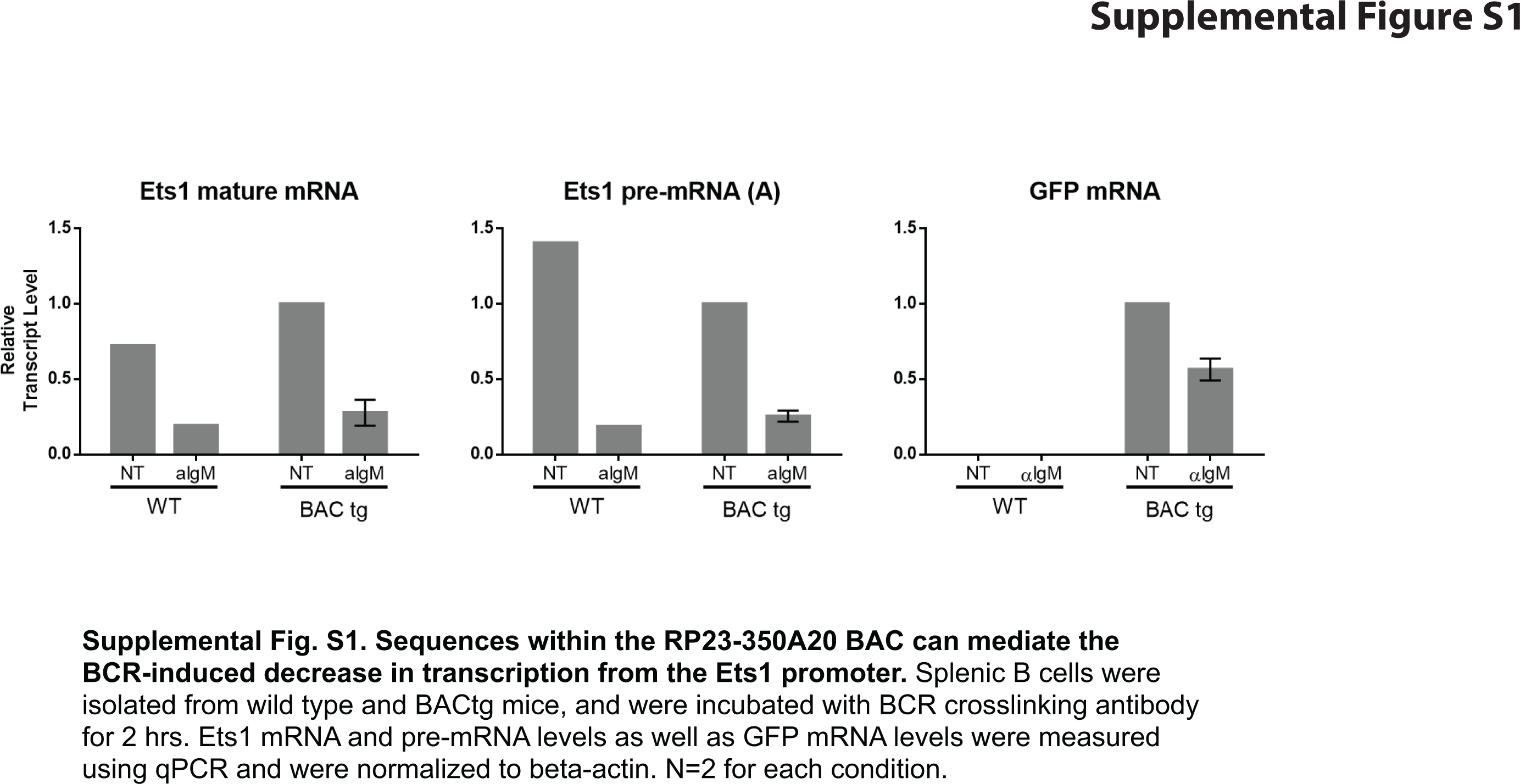

Supplement: S1 Fig — Splenic B cells were isolated from wild type and BACtg mice, and were incubated with BCR crosslinking antibody for 2 hrs. Ets1 mRNA and pre-mRNA levels as well as GFP mRNA levels were measured using qPCR and were normalized to beta-actin. N = 2 for each condition. (TIF) [file pone.0308896.s001.tif]

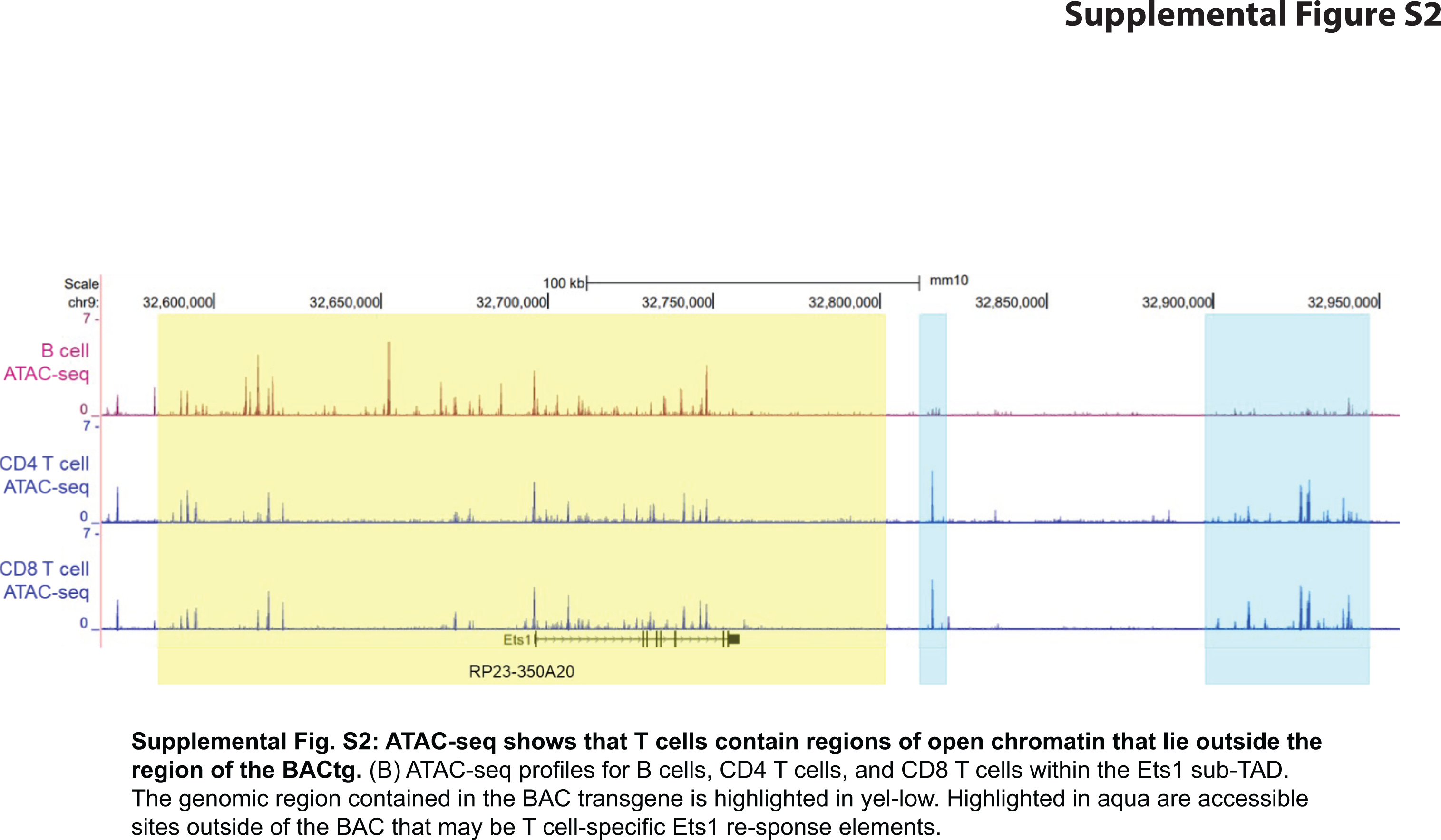

Supplement: S2 Fig — (B) ATAC-seq profiles for B cells, CD4 T cells, and CD8 T cells within the Ets1 sub-TAD. The genomic region contained in the BAC transgene is highlighted in yellow. Highlighted in aqua are accessible sites outside of the BAC that may be T cell-specific Ets1 response elements. (TIF) [file pone.0308896.s002.tif]

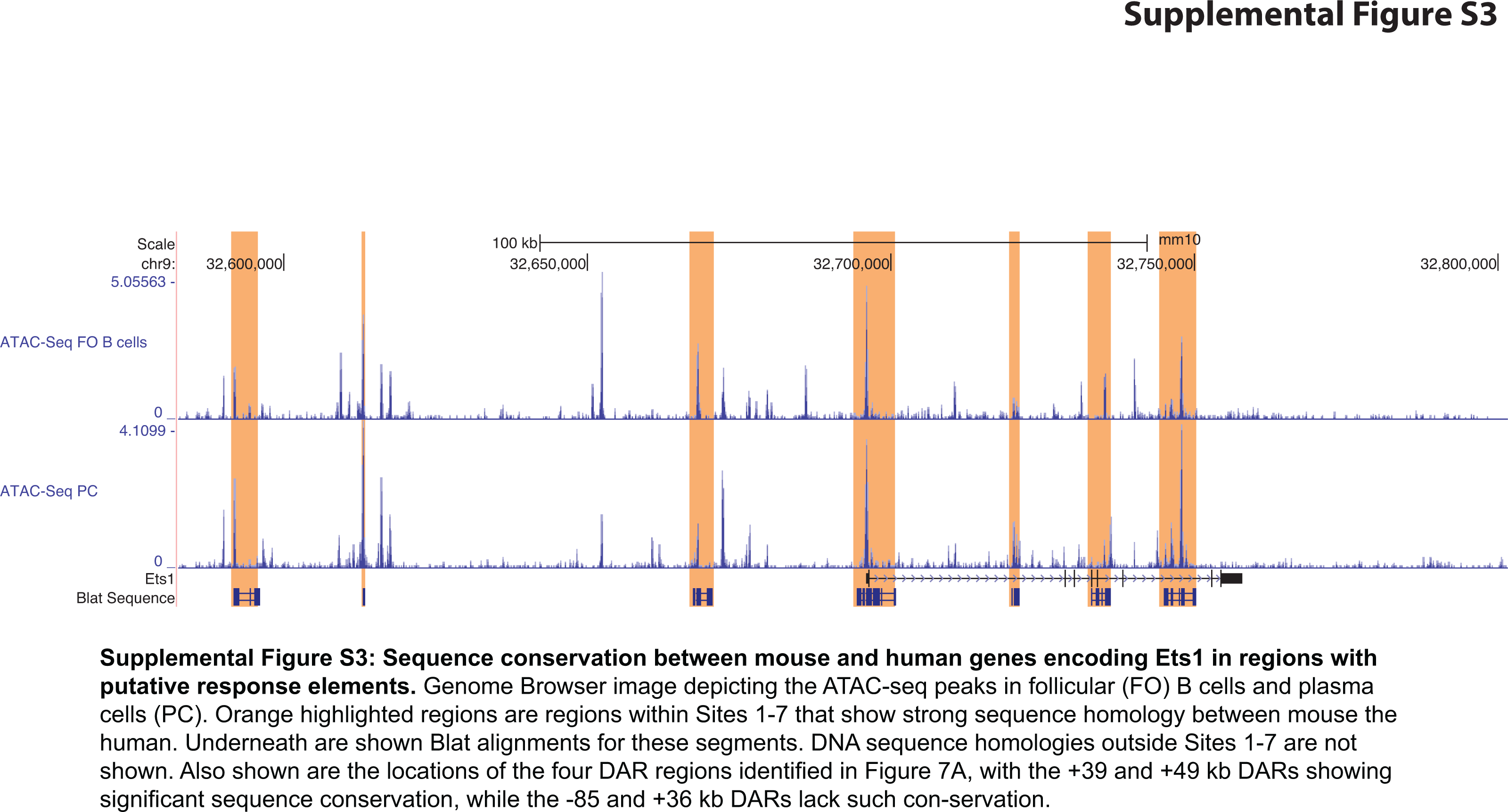

Supplement: S3 Fig — Genome Browser image depicting the ATAC-seq peaks in follicular (FO) B cells and plasma cells (PC). Orange highlighted regions are regions within Sites 1-7 that show strong sequence homology between mouse and human. Underneath are shown BLAT alignments for these segments. DNA sequence homologies outside Sites 1-7 are not shown. Also shown are the locations of the four DAR regions identified in Fig 7A, with the + 39 and + 49 kb DARs showing significant sequence conservation, while the -85 and + 36 kb DARs lack such conservation. (TIF) [file pone.0308896.s003.tif]

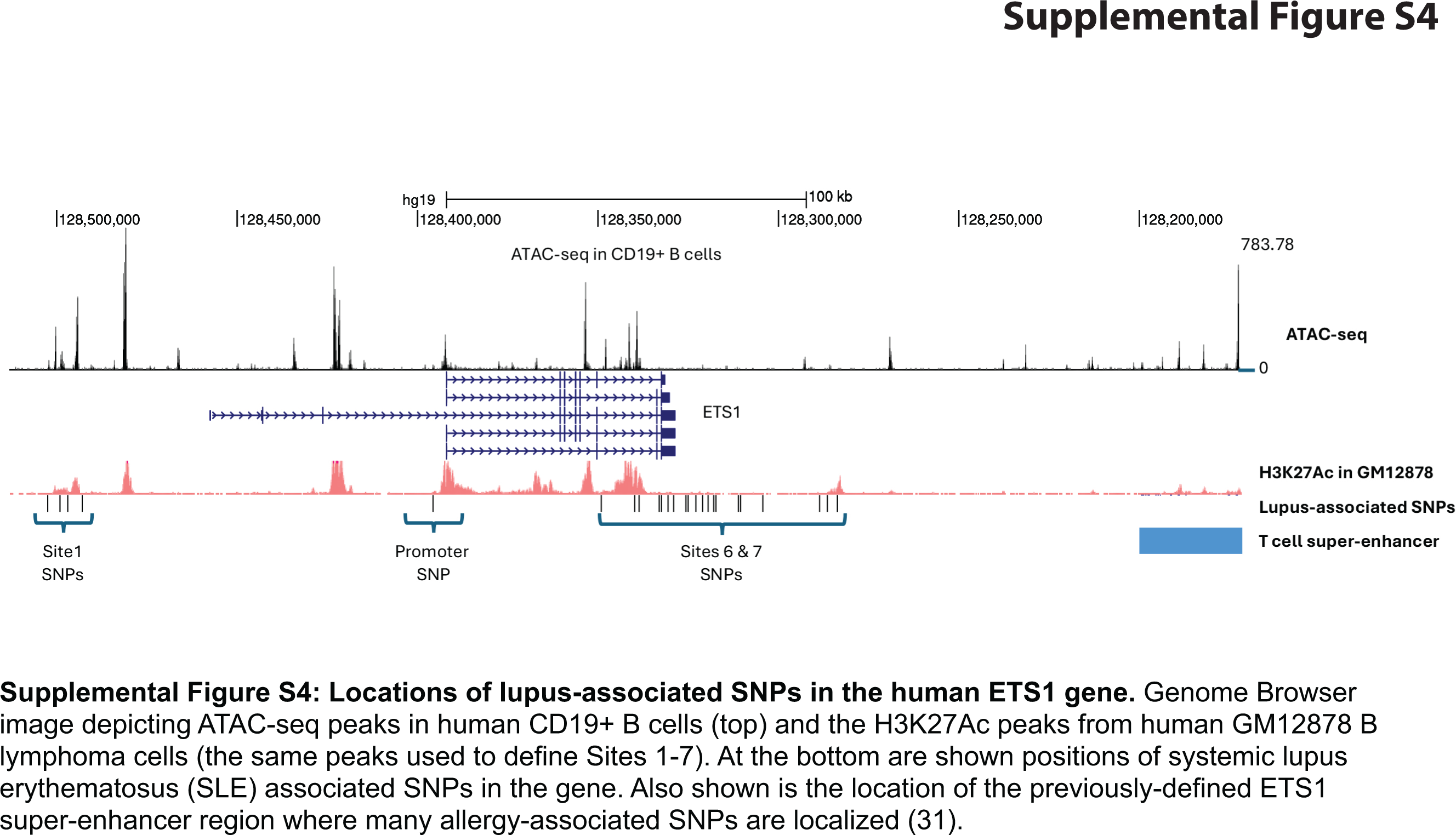

Supplement: S4 Fig — Genome Browser image depicting ATAC-seq peaks in human CD19 + B cells (top) and the H3K27Ac peaks from human GM12878 B lymphoma cells (the same peaks used to define Sites 1-7). At the bottom are shown positions of systemic lupus erythematosus (SLE) associated SNPs in the gene. Also shown is the location of the previously-defined Ets1 super-enhancer region where many allergy-associated SNPs are localized [31]. (TIF) [file pone.0308896.s004.tif]
