# Supplementary material for: Sequences within and upstream of the mouse Ets1 gene drive high level expression in B cells, but are not sufficient for consistent expression in T cells
Source: PLoS One. 2025 Mar 7;20(3):e0308896. doi: 10.1371/journal.pone.0308896 (PMC11888140; doi:10.1371/journal.pone.0308896)
Supplement: Attachment — Submitted filename: Response to Reviewer.pdf [file pone.0308896.s006.pdf]

Response to Reviewer Comments

Reviewer #1: In this paper, the authors set out to establish the regulatory regions that control Ets1 expression in B and T cells. Through combining chromatin state mapping and BAC transgenesis, they identify a region responsible for Ets-1 expression in B cells and establish that additional sequences outside this region are required for robust expression in T cells. The latter are not pursued. Functionally, the authors test various potential regulatory regions for enhancer activity but none are obviously active in that context. Furthermore, motif analysis identifies potential TF regulators but no experiments are provided to test their functionality in B cells. Overall, this study therefore contributes to our understanding of Ets1 expression control in immune cells but is rather preliminary in nature, stopping short of providing any detailed mechanistic insights rather than the current inferences.

Generally, the data as presented are okay but there are a few places for improvement:
(1) As indicated above, some functional testing of potential TF regulators would be a step forwards. (2) As the luciferase reporter assays are generally none informative, a better strategy would be to engineer changes in the regulatory regions either in the context of the BAC or via CRISPR in appropriate cells line models.

We agree with the reviewer that further studies are needed to identify relevant transcription factors and enhancer sequences needed for B cell expression. In this regard, Our lab has shown that signaling via the kinase IKK is required to downregulate Ets1 expression in response to BCR or TLR activation in B cells, although individually neither RelA nor cRel was required (see our published manuscript – PMID: 36445360). We also found that Foxo3a was not required for either the basal expression of Ets1 nor its downregulation in response to BCR ligation. We are continuing to perform follow-up experiments to examine roles for additional transcription factors (Pax5, Irf4, Ebf1, Bach2, PU.1 and Foxo1) that are expressed at high levels in resting B cells and that might be involved in regulating the basal levels of Ets1, but these studies have yet to yield conclusive results. We posit that combinatorial actions of transcription factors are likely to be at play, just as the case with the enhancers that we report in this study

The reviewer brings up a valid point about the limitations of the transient transfection system with luciferase reporter genes. We are currently developing Crispr systems to mutate putative enhancers in a B cell line. However, these studies are at a very early stage and we are planning to include them in a future publication examining Ets1 regulation in more detail.

Despite the fact that the studies reported in this manuscript have not yet identified a specific master transcription factor or enhancer segments crucial for regulating Ets1 expression, we feel that our studies still add valuable information to the knowledge of regulatory mechanisms that govern Ets1 expression. We have found that the Ets1 gene is regulated differently in B cells and T cells, something that was previously unknown. We have also found that sequences within and upstream of Ets1 are sufficient both for faithful expression in B cells, but also for stimulation induced decrease in Ets1 transcription as well as the decrease that occurs in plasma cells. We have also identified potential regulatory regions for Ets1 as marked by ATAC-seq peaks and histone marks. We have identified changes in chromatin accessibility that occur in B cells in response to BCR ligation – this experiment being a genome-wide study offers a valuable resource. While we have focused on describing such changes in the Ets1 locus, our ATAC-seq data have been submitted to the GEO databank and are available for other researchers to examine when assessing how BCR signaling alters chromatin in various gene loci.

Finally, it is also important to note that journal information for PLoS One states that "We evaluate research on the basis of scientific validity, strong methodology, and high ethical standards—not perceived significance. Multidisciplinary and interdisciplinary research, replication studies, negative and null results are all in scope". We feel that our manuscript currently meets this criterion for publication.

(3) In Fig. 4B, adding the human data is okay but the rationale in the text is not really correct. i.e. this does not allow "better definition of the regions" but instead provides evidence for evolutionary conservation.

We thank the reviewer for their thoughtful insights. We have modified this section of the text to better reflect the rationale.

(4) In Fig. 5B, the second track should be labelled as Fo B cells. Also, what population of B cells does the bottom track come from?

We have relabeled the figure. The bottom track in this figure is from data collected as part of this study (PMID: 25103404). It represents bulk CD3-, B220+, CD19+ B cells isolated from mouse spleen, which has been noted.

(5) In Fig. 6C, the percentage of regions containing each motif should be added.
In the left table, the authors should comment on the second motif that resembles at ETS motif. On the right side, they cannot conclude this is Ets1 rather than another ETS TF, therefore the results text describing this needs rewording.

These motifs were discerned using the motif-finding algorithm HOMER (http://homer.ucsd.edu/homer/introduction/basics.html). HOMER scores motifs by looking for sequences with differential enrichment between two sets of sequences. In our case these two sets are DAR sequences from unstimulated versus stimulated B cells. HOMER

calculates a p value for enrichment of known transcription motifs, but does not provide a value for the percentage of sequences containing a particular motif. Given the very small p values of the listed transcription factors, it is likely that they are strongly enriched.

To identify potential transcription factors binding to the enriched sequences, HOMER uses the TRANSFAC database. TRANSFAC contains experimentally-determined motifs for many transcription factors including various members of the Ets gene family. While the core Ets binding motif is similar among the various Ets family members, the flanking sequences are somewhat different. Using the TRANSFAC database, HOMER found that the best match to the enriched sequence was the Ets1 binding motif. However, we do agree that other related transcription factors in the Ets gene family may also bind to this sequence. For that reason, we have revised the text to include this point.

(6) I was confused by Fig. 8C. It appears here that several regions seem to be repressive in nature (ie less than promoter alone), so not sure why the results text says that none of the regions show strong repressive activity.

The reviewers is correct and we have revised the text accordingly.

(7) Given that GWAS signals suggest the importance of the Ets1 locus, do any of the SNPs map to putative regulatory elements highlighted in this study? If so what is their predicted effect on activity (ideally would also be tested but as a minimum discussed).

Several disease-associated SNPs do map close to or within putative enhancer regions. We've included a new figure showing where the SNPs associated with lupus map (new Supplemental Figure S5).

(8) *Additional information for reviewer:*

It should also be noted that some changes have been made to format the revised manuscript according to guidelines for PLoS One. As part of this effort, we have moved the figure legends into the main body of the text just under the paragraph in which they are first mentioned. This is consistent with the recommended formatting style for this journal - https://journals.plos.org/plosone/s/file?id=wjVg/PLOSOne_formatting_sample_main_body.pdf. The Supplemental Figure Legends have been moved into the figures themselves.
