# Supplementary material for: Sequences within and upstream of the mouse Ets1 gene drive high level expression in B cells, but are not sufficient for consistent expression in T cells
Source: PLoS One. 2025 Mar 7;20(3):e0308896. doi: 10.1371/journal.pone.0308896 (PMC11888140; doi:10.1371/journal.pone.0308896)
Supplement: Attachment — Submitted filename: Response to Reviewer.docx [file pone.0308896.s007.docx]

**Response to Reviewer Comments**

**Reviewer #1:** Most things have been addressed.

I appreciate this is PLOSONE and there are criteria for publication, and my initial comments were based on making the paper more conclusive rather than suggestive. I do take the arguments they make but it would be useful to add caveats about the limitations of the approach (ie surrounding previous points 1 and 2 about more appropriate definitive assays).

*We have added some sentences to address this on pages 22-23 of the revised paper.*

One this not addressed was "In Fig. 6C, the percentage of regions containing each motif should be added.

*While we would like to address this point, unfortunately the software used to identify motifs (HOMER software - http://homer.ucsd.edu/homer/) does not provide the percentage of sequences that contain a motif. It simply identifies motifs that are over-represented in one dataset (e.g., stimulated B cells) as compared to the other dataset (e.g., unstimulated B cells) and gives a p value for the significance of the over-representation. The p values for the motifs we list are all very small, indicating that these sequences are highly enriched in one dataset or the other. But we cannot provide a percentage of sites that contain those particular motifs.*

In the left table, the authors should comment on the second motif that resembles at
ETS motif. " The authors have not commented on the ETS-like motif. For the percentage of regions with motifs issue, then this is still useful to include as it provides a feel for what proportion of the effect is driven by a particular TF. You can get highly enriched regions in a low percentage of regions if the genomic back ground is also low for example. Again, this is not essential but just helps the reader to gauge the influence of ETS TFs.

*We agree with the reviewer’s point, but unfortunately as described above, we have no information on the percentages of sequences with a particular motif.*

new Supplemental Figure S5 was not included or referred to in the revised manuscript so was impossible to evaluate.

*Sorry, this was a mistake on our part. The extra figure is Supplemental Figure 4, which was included in the figures along with a figure legend, but was not referenced in the text. We have added sentences on page 14 of the manuscript to refer to the new figure.*
